# Adenine-functionalized Spongy Graphene for Green and High-Performance Supercapacitors

**DOI:** 10.1038/srep43104

**Published:** 2017-02-20

**Authors:** Dalia M. El-Gendy, Nabil A. Abdel Ghany, E. E. Foad El Sherbini, Nageh K. Allam

**Affiliations:** 1 Energy Materials Laboratory (EML), School of Sciences and Engineering, The American University in Cairo, New Cairo 11835, Egypt; 2 Physical Chemistry Department, National Research Centre, Dokki, Giza, Egypt; 3 Chemistry Department, Faculty of Science, Ain Shams University, Cairo, Egypt

## Abstract

A simple method is demonstrated to prepare spongy adenine-functionalized graphene (SFG) as interconnected, porous 3-dimensional (3D) network crinkly sheets. Such 3D network structure provides better contact at the electrode/electrolyte interface and facilitates the charge transfer kinetics. The fabricated SFG was characterized by X-ray diffraction (XRD), FTIR, scanning electron microscopy (FESEM), Raman spectroscopy, thermogravimetric analysis (TGA), UV−vis absorption spectroscopy, and transmission electron microscopy (TEM). The synthesized materials have been evaluated as supercapacitor materials in 0.5 M H_2_SO_4_ using cyclic voltammetry (CV) at different potential scan rates, and galvanostatic charge/discharge tests at different current densities. The SFG electrodes showed a maximum specific capacitance of 333 F/g at scan rate of 1 mV/s and exhibited excellent cycling retention of 102% after 1000 cycles at 200 mV/s. The energy density was 64.42 Wh/kg with a power density of 599.8 W/kg at 1.0 A/g. Those figures of merit are much higher than those reported for graphene-based materials tested under similar conditions. The observed high performance can be related to the synergistic effects of the spongy structure and the adenine functionalization.

Supercapacitors have attracted much attention lately due to their high power density, reversibility, and long cycle life^1^. According to their charge storage mechanism, supercapacitors can be classified into two basic types; electric double-layer capacitors (EDLCs) and pseudocapacitors. While the EDLCs store energy physically by charge accumulation at the electrode/electrolyte interface^2^, the battery-type or pseudocapacitors store energy chemically by fast and reversible Faradic reactions in the electrode materials^3^. The pseudocapacitive materials such as metal oxides^4^ and conducting polymers^5^ can achieve relatively higher capacitance than EDLCs. However, they are limited by poor cyclability due to the structural degradation of the electrodes during the redox processes^6^. Therefore, carbonaceous materials loaded with metal oxides or polymers are widely investigated as alternatives^7^,^8^,^9^, including carbon nanotubes (CNTs), activated carbons (AC), graphite, graphene oxide, and graphene. These materials have been actively used due to their abundance, ease of fabrication, and wide working temperature range. Different modifications have been applied to those materials to increase their surface area and tailor their pore size distribution (PSD), resulting in an improvement of energy and power operation parameters of the capacitors^8^,^9^.

Specifically, graphene-based materials are given much consideration as effective electrode materials owing to their high specific surface area, excellent chemical stability, electrical and mechanical properties, and the feasibility for large-scale production of chemically-modified graphene (CMGs)^10^,^11^,^12^. To this end, the Hummers’ method is widely used to produce graphene oxides (GO)^13^. However, the poor electrical conductivity of GO hinders efficient charge transfer and reduces cyclability^13^,^14^. These drawbacks can be overcome by partially restoring the graphene sp^2^ network via chemical reduction or by thermal annealing to partly remove the O-containing groups^15^,^16^. This is not without drawbacks as the synthesis of graphene using reducing agents (e.g., hydrazine, dimethyl hydrazine, and NaBH_4_) is harmful to the environment and the resulted graphene has a strong tendency to restack owing to the π–π interactions^17^,^18^,^19^,^20^. Therefore, an easy, eco-friendly method to reduce GO is urgently needed.

Herein, a simple method is demonstrated to prepare spongy functionalized graphene (SFG) *via* freeze-drying graphene oxide solution to prevent graphene sheets from restacking together, resulting in interconnected, porous 3D network crinkly sheets. Such 3D network structure provides better contact at the electrode/electrolyte interface and facilitates the charge transfer kinetics. This was followed by functionalization of the graphene oxide with adenine. The functional groups facilitate the interaction between the host materials by condensation and nucleophilic addition reactions between –NH_2_ groups of adenine and carboxylic acid or epoxy groups on the GO, leading to various changes of the graphene structure such as increasing the interlayer spacing or layer scrolling. After annealing, functionalized spongy graphene architecture (FSG) was obtained, which helps to prevent the stacking between graphene interlayers. The FSG electrodes showed high performance upon their use in electrochemical capacitor assembly.

## Experimental methods and Materials

### Materials

Graphite powder (<20 μm) and Nafion 117 solution (5%) were purchased from Sigma Aldrich. Sulfuric acid (H_2_SO_4_, 99%) from Sham lab., Hydrogen peroxide (H_2_O_2_, 30% W/V) from LOBA Chemie, Absolute ethanol and HCl (33%) were purchased from El-Nasr Pharmaceutical Company, Egypt. Finally, potassium permanganate (KMnO_4_, 99%), from Arabic Laboratory Equipment Co., and Adenine (Merck) were used directly without further purification. Distillated water was used for washing the products.

### Synthesis of spongy graphene oxide (SGO)

GO was prepared from natural graphite using a modified Hummers’ method^21^. In a typical experiment, graphite (1.5 g), NaNO_3_ (1.5 g) and H_2_SO_4_ (70 ml) were mixed and stirred in an ice bath. Subsequently, 9 g of KMnO_4_ were added slowly. The reaction mixture was warmed to 40◦C and stirred for 1 h. Water (100 ml) was then added and the temperature was increased to 90◦C for 30 min. Finally, 300 ml of water were added slowly, followed by the slow addition of 10 ml of 30% H_2_O_2_. The reaction mixture was filtered and washed with 0.1 M HCl and water. The GO precipitate was dispersed in a water/methanol (1:5) mixture and purified with three repeated centrifugation steps at 10000 rpm for 30 min. The purified sample was dispersed in deionized water and centrifuged at 2500 rpm and finally washed with deionized water and sonicated for 1 hour to obtain highly exfoliated graphene oxide. The GO precipitate was dispersed in water/methanol mixture and purified with repeated centrifugation steps at 10000 rpm for 30 min. Note that washing with 0.1 M HCl and water resulted in highly exfoliated GO sheets. To prepare SGO, GO solution (4 mg/l) was frozen at −18 °C for 2 days. Subsequently, the tube was peregrinated to a freeze-dryer and dried at a temperature of −53 °C and a pressure of 10 Pa for 3 days^22^, see Scheme 1.

### Preparation of adenine-functionalized graphene oxide (FGO)

**Figure 1:**
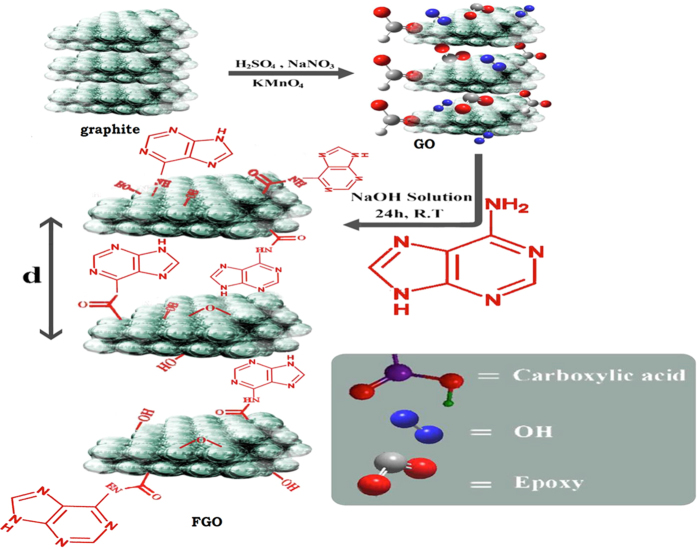
Synthesis of GO and adenine-functionalized grapheme.

GO (0.1 g) was dispersed in distilled water (10 ml), then (0.3 g) adenine and equimolar amount of NaOH in distilled water (10 ml) were added. The mixture was stirred for 24 h. The resulted precipitate was centrifuged, washed well with water/ethanol mixture and finally dried at 60 °C^23^, see Fig. 1.

### Preparation of reduced graphene oxide (RGO) and functionalized graphene (FG)

The Reduced graphene oxide was prepared by the hydrothermal reduction method. Briefly, 25 mg of graphene oxide powder was added to a 100 ml conical flask containing 40 ml of deionized water and sonicated for 30 min to obtain a homogeneous dispersion. For the functionalized graphene, 25 mg of FGO powder was added to a 100 ml conical flask containing 40 ml of deionized water and sonicated for 30 min to obtain a homogeneous dispersion. The solution was transferred to a Teflon-lined autoclave and heated at 170 °C for 8 h, and then left to cool at room temperature to get black product. This product was washed several times with deionized water and collected through centrifugation. Finally, FG was dried in an oven at 60 °C.

### Preparation of electrodes and electrochemical measurements

Glassy carbon (GC) electrode (5.0 mm diameter) was polished with alumina nanopowder and rinsed with deionized water. Fresh dispersion of the sample was prepared for each experiment by dispersing 5.0 mg of the FG powder in 0.5 ml Nafion 117 solution (1%) by ultra-sonication for 30 min. Then, 10 μl suspension of the material was cast onto the surface of the electrode with a micropipette, which accounts for a loading of 0.1 mg. Finally, the working electrode was dried at 60 °C for 10 min and left to cool down.

All the electrochemical measurements were performed in a three-electrode system, where the working electrode was made of a glassy carbon disk, the standard calomel electrode (SCE) and platinum wire were used as reference and counter electrodes, respectively. The electrochemical measurements were carried out in 0.5 M H_2_SO_4_ using Auto lab-302N electrochemical workstation (Metrohm). The cyclic voltammetry (CV) measurements were done in the potential range −0.2 to 1 V at various scan rates (1–200 mV/s). Galvanostatic charge/discharge measurements were run from −0.2 to 1 V at current densities of 1, 2, 3, 4, 5 A/g.

### Characterization techniques

The crystal structure of the prepared materials was examined by X-ray diffraction (XRD, XPERT- PRO- Analytical) with Cu Kα radiation (λ = 1.54 °A). The surface morphology was investigated by field-emission scanning electron microscope (FESEM-Zeiss SEM Ultra-60). The morphology of the samples was investigated using high-resolution transmission electron microscope (HRTEM, JOEL JEM-2100) operating at an accelerating voltage of 120 kV. The infrared (IR) spectra were recorded using a JASCO spectrometer (FTIR-6300 type A) in the range 400–4000 cm^−1^. The UV/Vis spectrophotometric measurements were made using a Shimadzu 2040 spectrophotometer. Raman measurements were performed using a micro-Raman microscope with an excitation laser beam wavelength of 325 nm. The weight loss of the samples was determined using TGA thermal analyzer (TA TGA-Q500) from room temperature to 800 °C at a heating rate of 10 °C/min in nitrogen atmosphere.

## Results and Discussion

**Figure 2:**
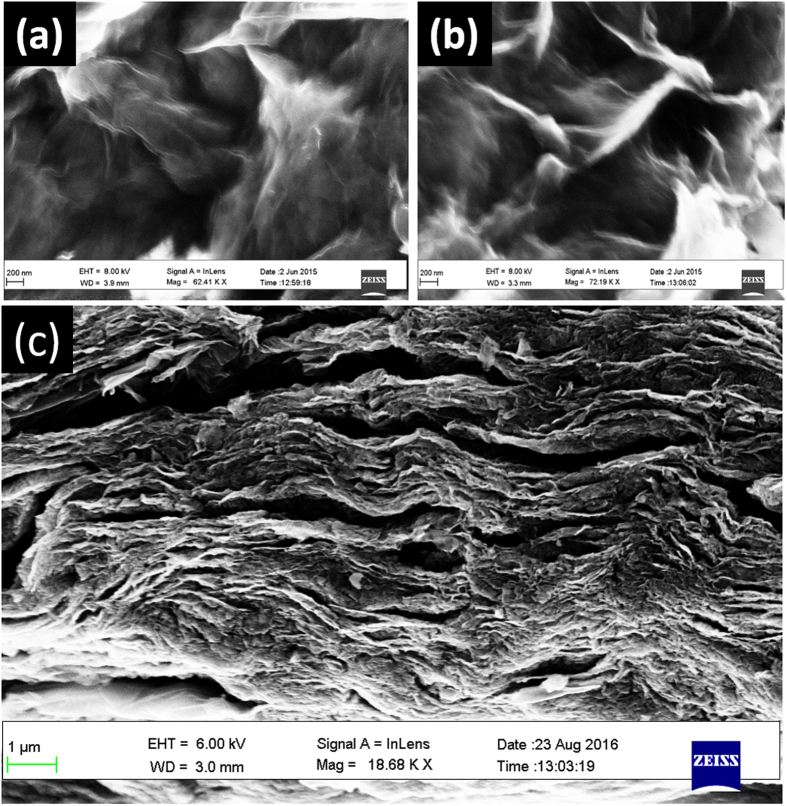
FESEM Images of (**a**) spongy GO, (**b**) FGO and (**c**) FG.

Figure 2 shows FESEM images of the fabricated materials. Figure 2a depicts the surface of the fabricated spongy graphene oxide (SGO), revealing a large increase in the thickness of the layers, which is possibly due to the formation of oxygen groups in the basal plane of graphene. Upon adding adenine, Fig. 2b, the graphene becomes more exfoliated with further increase in volume, resulting in flake-like structure with wrinkled edges and crumble graphene structure. Figure 2c shows the morphology of the synthesized functionalized-graphene (FG), exhibiting interconnected, porous 3D framework of randomly oriented, crinkly sheets. Those wrinkles clearly act to prevent graphene sheets from restacking.

**Figure 3:**
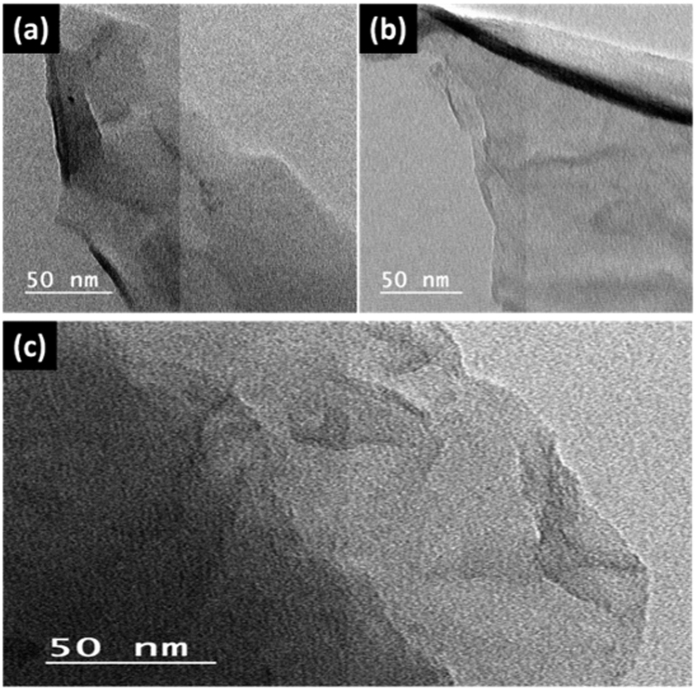
TEM images of (**a**) spongy GO, (**b**) FGO, and (**c**) FG.

Figure 3 shows the corresponding TEM images, where the GO sheets (Fig. 3a) appeared crumpled with lots of folds. The TEM image of the FGO (Fig. 3b) shows a smooth and transparent surface. The exfoliated crumpled thin flake with its wrinkled structure is due to the presence of adenine between the layers after covalent functionalization. Figure 3c shows the TEM image of FG. Note that the morphology remained the same after the hydrothermal treatment.

**Figure 4:**
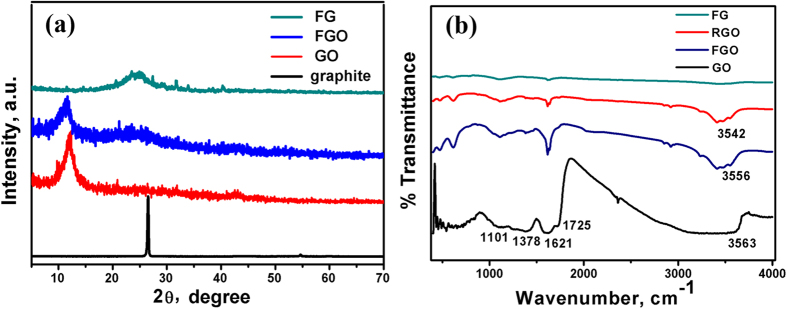
(**a**) XRD pattern and (**b**) FTIR spectra of the pristine graphite, GO, FGO and FG.

Figure 4a illustrates the XRD pattern of pristine graphite, GO, FGO and FG. The peak appeared in the diffraction pattern of graphite at 2ϴ = 26.5° corresponds to c-axis and reflection from the (0 0 2) plane with interlayer spacing of 0.34 nm. The spectra of GO is a single and sharp diffraction peak at 2ϴ = 12° with an interlayer spacing of 0.83 nm, suggesting that the GO is devoid of any graphite^24^. The increase in the d-value from 0.34 to 0.83 nm is due to the increase of the interlayer spacing along the c-axis, which can be attributed to the presence of oxygen atoms on the GO sheets^25^. The diffraction pattern of the adenine-functionalized GO exhibits both increasing and decreasing in the interlayer spacing. As a result of π-π stacking and H-bonding interactions, the crystallinity of graphene decreases and becomes somewhat amorphous. These factors enhanced the attraction between adjacent layers, resulting in an irreversible aggregation or even restacking to graphite. Upon reducing the GO via hydrothermal treatment, the sharp diffraction peaks at 24.7 °and 12° disappeared, supporting the formation of graphene sheets (FG) and complete reduction of GO.

Due to the physical damage of its conjugated structure as a result of the treatment with strong acids, graphite oxide is insulating in nature. Figure 4b shows the FTIR spectra of GO, FGO, RGO and FG. Note the several peaks for various functional groups that were identified in the FTIR spectrum of GO: the broad band centered around 3563 cm^−1^ can be assigned to O-H stretching vibrations ν(OH_2_) attributed to adsorbed water. The other bands appeared at 1725 cm^−1^ can be attributed to the stretching vibrations ν(C = O) of COOH group corresponding to carbonyl and carboxyl groups, the band at 1621 cm^−1^ attributed to in-plane vibration (C = C) from un-oxidized sp^2^ CC bonds, the intense band at 1378 cm^−1^corresponding to O-H deformation of C–OH group, and the band at 1101 cm^−1^ attributed to ν(C–O) stretching vibrations mode^26^. The peak at 1725 cm^−1^ almost disappears and the peak emerging at 1637 cm^−1^ is characteristic of the C = O stretching in the amide group, which cannot be found in the spectrum of GO. Stretching of the amide C−N appears as a strong peak at 1188 cm^−1^. The peaks at 1560 and 1618 cm^−1^ are attributed to the graphene vibration, and the peak attributed to the OH and NH stretching groups at 3475 cm^−1^confirm the covalent functionalization of the neat graphite by adenine, assuring the successful functionalization process. The peak at 3415 cm^−1^ is attributed to N−H stretching^27^. These results demonstrate that adenine molecules were covalently bonded to GO by the amide linkage. Note that the peak intensity of the C-O and C-O-C (epoxide) groups, respectively, decreased in RGO, FGO, and after the hydrothermal reduction that resulted in FG.

**Figure 5:**
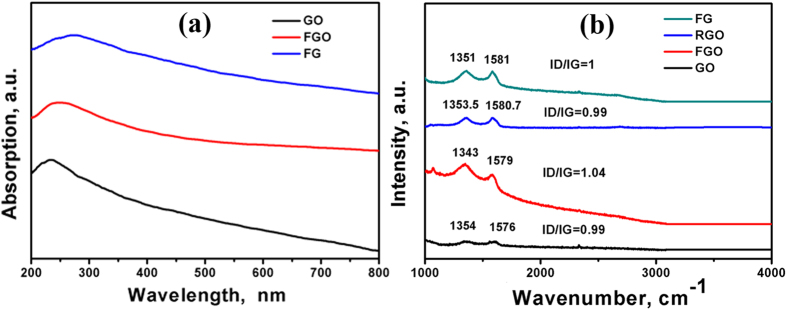
(**a**) UV-Vis absorption and (**b**) Raman spectra of GO, FGO and FG.

Figure 5a illustrates the UV-Vis absorption spectra of the synthesized materials. GO exhibits a strong peak centered at 236 nm with an extended shoulder at 300 nm, corresponding to π-π* transitions of aromatic C = C band and n-π* transitions of C = O band, respectively^28^. Upon functionalizing GO with adenine to form FGO, the golden brown color of GO solution turned to dark brown for FGO due to the partial deoxygenation during functionalization^28^. This results in a red-shift in the absorption peak to 246 nm, which can be related to the partial restoration of electronic conjugation in the aromatic carbon structure. The hydrothermal treatment results in simultaneous reduction of FGO to form the FG. The reduction of FGO was confirmed by the UV–Vis spectra, where FGO underwent partial deoxygenation, which resulted in the red-shift of the absorption peak^28^ from 246 nm to 270 nm.

Raman spectroscopy is a powerful technique to investigate the structure and quality of carbon-based materials. Figure 5b shows the Raman spectra for GO, FGO, RGO and FG. The intensity ratio of the D and G bands (I_D_/I_G_) is a useful parameter for determining the sp^2^ domain size of a carbon structures containing sp^3^ and sp^2^ domains^29^. GO exhibited the G band at 1576 cm^−1^ and the D band at 1354 cm^−1^. While the intensity of the D band for GO was increased compared to that of graphite, the G band is still prominent and the I_D_/I_G_ ratio is 0.99. Upon functionalization of GO with adenine, the G and D bands are shifted to 1579 and 1343 cm^−1^, respectively and the D band becomes more prominent. The higher I_D_/I_G_ ratio of FGO (1.04) than that of GO (0.99), indicates the introduction of sp^3^ domain upon functionalization of GO with adenine. RGO exhibited a G band at 1580.7 cm^−1^ and a D band at 1353.5 cm^−1^, respectively and FG exhibited the G band at 1581 cm^−1^ and the D band at 1351 cm^−1^, respectively, with the D band becoming more prominent. However, the I_D_/I_G_ ratio of FG is 1.0, slightly smaller than that of FGO (I_D_/I_G_ = 1.04). Therefore, it can be deduced that the extensive oxidation and solvothermal reduction decreased the in-plane sp^2^ domains and increased the edge planes, as well as the disorder in the prepared FG^29^.

**Figure 6:**
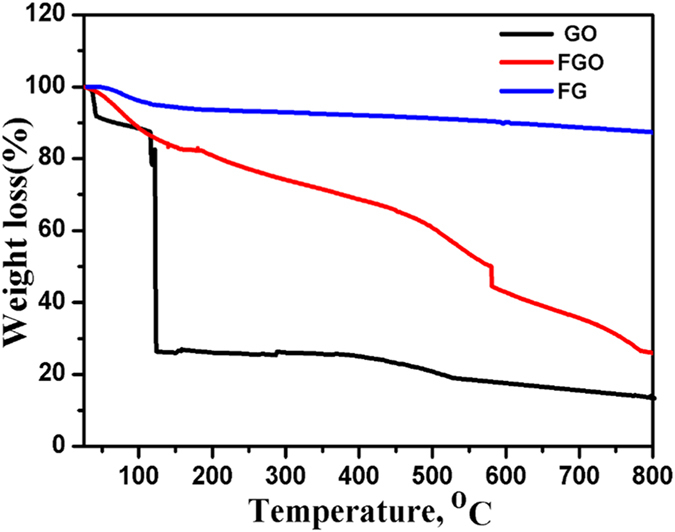
TGA analysis for GO, FGO and the FG.

To assess the thermal stability of GO, FGO and FG, TGA analysis was performed by heating the material under nitrogen atmosphere to 800 °C at a rate of 10 °C/min, Fig. 6. At temperatures below 100 °C, the mass loss can be related to the removal of adsorbed water. GO is thermally unstable and losses weight in three stages: the first stage is observed below 110 °C that can be related to moisture content and evaporation of interstitial H_2_O^30^ with a total mass loss of ~8%. The second stage is observed around 130–250 °C as a sharp drop peak that accounts for mass loss of~ 65%, which is coming from the decomposition of hydroxyl groups, intercalated water on GO, and carboxyl group to produce gases like H_2_O and CO_2_. Note that CO_2_ is usually produced from the decomposition of carboxyl group as a result of thermal treatment at temperatures less than 500 °C. The third stage started from 350 °C up to 800 °C and the maximum mass loss reached about 80%, which can be attributed to the decomposition of carbonyl group formed on the surface of graphene oxide to produce CO gas as a result of thermal treatment at temperatures higher than 500 °C^31^. FGO showed higher thermal stability than GO. The first degradation step of FGO appeared almost in the similar range to that of GO (145–173 ◦C), whereas the second degradation step appeared at a much higher temperature of 365–415 ◦C. During the functionalization of GO with Adenine, the labile oxygen groups underwent chemical reaction to form a strong covalent bond with the amino groups of Adenine, which considerably decreased the amount of labile groups in FGO. Thus, very low weight loss was observed at around 145–173 ◦C in FGO. This result is also supported by the decrease in the FTIR peak intensity of the band at 1725 cm^−1^, which is characteristic of the stretching vibrations ν(C = O) of COOH group corresponding to carbonyl and carboxyl groups that have disappeared, and the peak emerging at 1637 cm^−1^ is characteristic of the C = O stretching in the amide group^32^, Fig. 4b. The weight loss of the hydrothermally reduced FG shows a slight a weight loss of about 10% up to 670 °C. These results indicate that the oxygen-based groups in GO have formed heat-stable structures via covalent bonding with the adenine.

**Figure 7:**
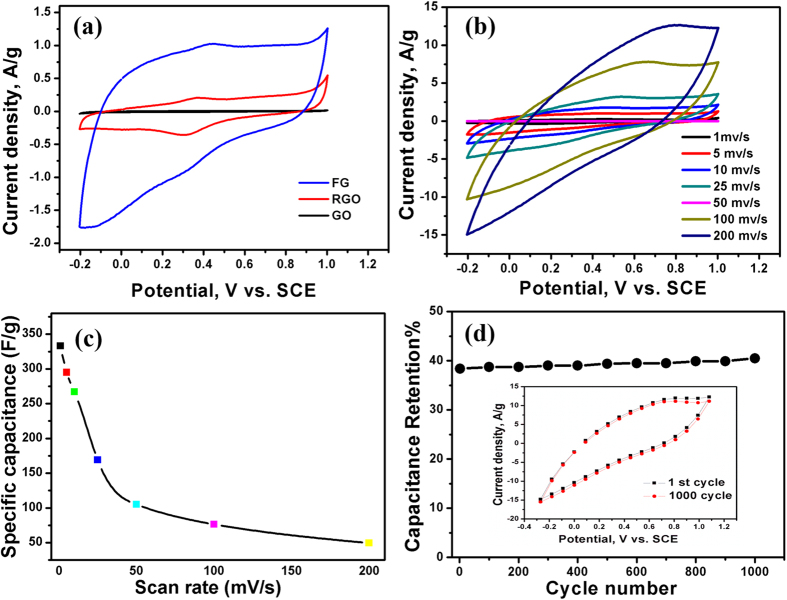
(**a**) Cyclic voltammogrames of FG, RGO and GO electrodes in 0.5 M H_2_SO_4_ at a scan rate of 5 mV/s, (**b**) the cyclic voltammogrames of FG electrodes at different scan rates, (**c**) the corresponding specific capacitance of FG electrodes at different scan rates in 0.5 M H_2_SO_4_, and (**d**) the first and 1000^th^ CV cycles of the FG electrodes.

To investigate the supercapacitive performance of the fabricated samples, cyclic voltammetry (CV) measurements were performed in 0.5 M H_2_SO_4_, where the specific capacitance of the electrodes was calculated using Eq. 1.


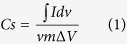


where *Cs* is the specific capacitance, *m* is the weight of the electrode (g), *I* is the response current density (A/g), *ΔV* is the potential difference, and *v* is the potential scan rate (mV/s). Figure 7a shows the cyclic voltammogrames of the GO, RGO and FG electrodes in 0.5 M H_2_SO_4_ at a scan rate of 5 mV/s. While the GO electrode shows negligible current response due to its insulating characteristics, the capacitance of the FG electrode is much higher than that of RGO (64.4 F/g) at a scan rate of 5 mV/s, which can be related to the presence of nitrogen atoms coming from adenine. The current of the FG electrode increases as the potential increases, suggesting an increasing electrical conductivity. The FG electrode shows quasi-rectangular CV curve. Irrespective of the scan rate, the FG electrode shows a weak cathodic (reduction) peak at ∼0.5 V_SCE_ and a corresponding anodic peak at ∼0.32 V_SCE_, Fig. 7b. The specific capacitance of the FG electrode reached up to 333 F/g at a scan rate of 1 mV/s and drops to 45.5 F/g at a scan rate of 200 mV/s, Fig. 7c. This is a common phenomenon as the ions in the electrolyte might not have enough time to enter into the complex micropores of the electrodes (diffusion limited) at high scan speeds. Note that the obtained specific capacitance of 333 F/g at a scan rate of 1 mV/s is much higher than that previously reported for graphene electrodes synthesized from graphite oxide by microwave (120 F/ g^33^, 191 F/g^34^) as well as other graphene reduced by different methods: hydrazine vapor-reduced graphene (155 F/g)^35^, graphene aerogels synthesized via the reduction of graphene oxide with L-ascorbic acid^36^, graphene oxide reduced using urea (255 F/g)^37^, electrochemically reduced graphene oxide in 0.05 M NaH_2_PO_4_ (223.6 F/g)^38^ and in 0.1 M KNO_3_ (158.5 F/g)^39^, and thermally-reduced the graphene oxide (260.5 F/g)^40^. Also, our obtained specific capacitance is much higher than that reported for exfoliated graphene (186.9 F/g) at a current density of 0.1 A/g^41^, nitrogen-modified graphene (227 F/g at 1 A/g)^42^, graphene made from natural graphite (170 F/g in H_2_SO_4_, 84 F/g in (C_2_H_5_)_4_NBF_4_/acetonitrile^43^, 80 F/g in a 1 M Na_2_SO_4 _44), and glutathione-functionalized graphene (238 F/g in 1 M H_2_SO_4_)^45^. Moreover, the FG electrodes exhibit excellent rate capability of 50.7% at a scan rate of 25 mV/s. The cycle life test of the FG electrode was performed at a scan rate of 200 mV/s for 1000 cycles, Fig. 7d. The specific capacitance sharply increased from the initial cycle until 1000 cycle to reach 102.2% of the initial cycle, indicating the excellent cycling stability of the FG electrodes.

**Figure 8:**
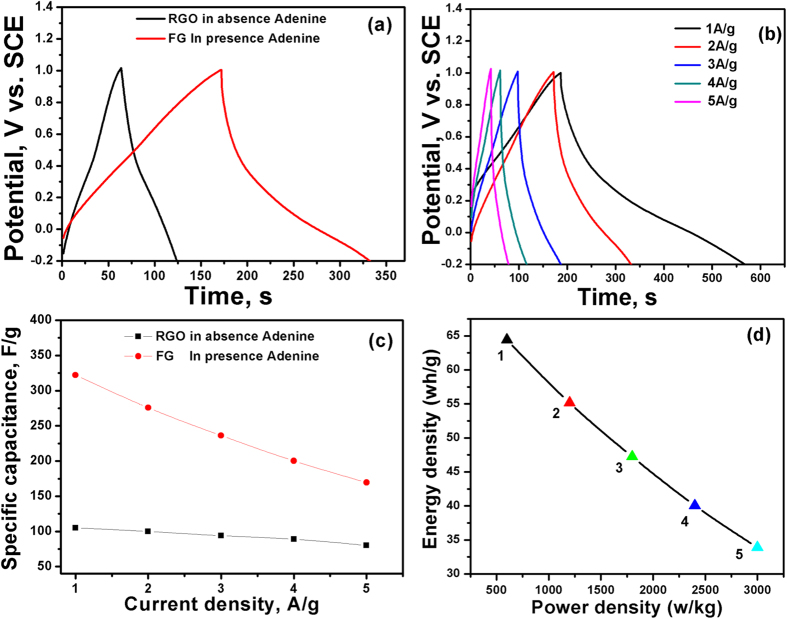
(**a**) Galvanostatic charge/discharge characteristics of the RGO and FG electrodes in 0.5 M H_2_SO_4_ at 2 A/g, (**b**) galvanostatic charge/discharge curves of FG electrodes at different current densities, (**c**) average specific capacitances of RGO and FG at various current densities, and (**d**) Ragone plot of FG electrodes at different current densities of 1, 2, 3, 4 and 5 A/g.

To extensively understand the electrochemical properties of the RGO and FG materials, the charge/discharge behavior was further examined by chronopotentiometry. The specific capacitance was calculated at different current densities using Eq. 2


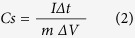


where *I* is the discharged current (A), *Δt* is the discharged time (s), and *ΔV* is the potential window (V). The charge/discharge capacitance (Fig. 8a) of the FG electrode at 2 A g^−1^ is 300 F g^−1^, which is three times higher than that exhibited by the RGO electrode (100 F g^−1^). Figure 8b shows the charge/discharge graphs of the fabricated spongy graphene electrodes at different current densities from 1 to 5 A/g. All the charge–discharge curves are quasi-triangular, which can be attributed to fast and capable charge transfer and high electrical conductivity^46^. This can be related to the existence of active nitrogen atoms from adenine and the high electronegativity of nitrogen that may generate dipoles on the surface of graphene^46^,^47^, which may attract charged species into the surface^48^,^49^. The effect of N and O atoms on the capacitance can be related to the inductive effect of the σ-bonded structure from N and O heteroatoms, which cause distribution of the electrons and polarization of some bonds, leading to reversible Faradic redox reactions^50^. The calculated specific capacitances were 322.1, 275.8, 236.2, 200.3 and 169.5 F/g at 1, 2, 3, 4 and 5 A/g, respectively. The specific capacitance at 1 A/g is calculated to be 322.1 F/g, which is lock to the data (333 F/g) derived from CV curve at scan rate 1 mV/s. Figure 8c depicts the variation of the specific capacitance with the current density. It is observed that there is a decrease in specific capacitance as the current density increases. Moreover, the FG electrodes exhibit excellent rate capability of 52.6% at 5 A.

The energy and power densities are very important performance metrics of supercapacitors, which can be calculated from the galvanostatic charge/discharge graphs using Eqs. 3 and 4.


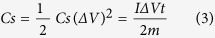



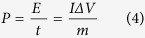


where E and P refer of the average energy density (Wh/Kg) and average power density (W/Kg), respectively, and Cs is the specific capacitance calculated from the charge/discharge curves, *I* is the discharge current (A), *t* is the discharge time (h), 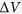 is the potential window (V), and *m* is the mass of the FG electrode (kg). Ragone plot for the FG electrode at different current densities is shown in Fig. 8d. The energy density can reach up to 64.42 Wh/Kg with a power density of 599.8 W/kg at 1.0 A/g. Note that 40.7 Wh/Kg and 2998.5 W/Kg remain there even at a current density as high as 5 A/g. It is worthy to mention that the obtained energy density for FG electrode 64.42 Wh/Kg) is much higher than those reported for thermally-reduced graphene (11.6 Wh/Kg)^51^, chemically-reduced graphene (11.5 Wh/Kg^52^, 5.8 Wh/Kg^53^), and graphene/onion like carbon composite electrodes (20 Wh/Kg)^54^.

## Conclusions

A green method is demonstrated to prepare 3-dimensional (3D) network crinkly sheets of adenine-functionalized spongy graphene (SFG), which provide better contact at the electrode/electrolyte interface and facilitate the charge transfer kinetics. The electron microscopy (FESEM and TEM) analysis showed the formation of exfoliated crumpled thin flake with wrinkled structure, which was attributed to the presence of adenine between the graphene layers after covalent functionalization. This was supported by the XRD analysis where the diffraction pattern of the adenine- functionalized graphene exhibits increasing in the interlayer spacing. Also, the FTIR spectra showed a peak at 3475 cm^−1^ that is attributed to the −OH and −NH stretching groups, confirming the covalent functionalization of the neat graphene by adenine. The higher I_D_/I_G_ ratio of FGO (1.04) than that of GO (0.99) indicates the introduction of sp^3^ domain upon functionalization of GO with adenine. However, the I_D_/I_G_ ratio of FG is 1.0, which is slightly smaller than that of FGO (I_D_/I_G_ = 1.04). The TGA analysis showed a slight weight loss of about 10% up to 670 °C for the FG, indicating that the oxygen-based groups in GO have formed heat-stable structures *via* covalent bonding with adenine. The synthesized materials have been evaluated as supercapacitor materials in 0.5 M H_2_SO_4_ using cyclic voltammetry (CV) at different potential scan rates, and galvanostatic charge/discharge tests at different current densities. The SFG electrodes showed a maximum specific capacitance of 333 F/g at a scan rate of 1 mV/s with excellent cycling retention of 102% after 1000 cycles at 200 mV/s. The energy density was 64.42 Wh/kg with a power density of 599.8 W/kg at 1.0 A/g. Those figures of merit are much higher than those reported for graphene-based materials tested under similar conditions. The observed high performance can be related to the synergistic effects of the spongy structure and the adenine functionalization.

## Additional Information

**How to cite this article:** El-Gendy, D. M. *et al*. Adenine-functionalized Spongy Graphene for Green and High-Performance Supercapacitors. *Sci. Rep.* **7**, 43104; doi: 10.1038/srep43104 (2017).

**Publisher's note:** Springer Nature remains neutral with regard to jurisdictional claims in published maps and institutional affiliations.
